# Case report: Polyarteritis nodosa as a substrate for a massive myocardial infarction

**DOI:** 10.3389/fcvm.2022.1070378

**Published:** 2023-01-12

**Authors:** Fabio Solis-Jimenez, Araceli Gonzalez-Ortiz, Juan H. Larios-Lara, Carlos A. Castro-Garcia, Eduardo I. Arteaga-Chan, Fernando Velazquez-Sanchez, Jorge L. Vargas-Estrada, Erika Y. Ramirez-Marcano, Diego Araiza Garaygordobil, Jose L. Briseño De La Cruz, Rodrigo Gopar-Nieto, Daniel Sierra-Lara Martinez, Alexandra Arias-Mendoza

**Affiliations:** ^1^Cardiology Department, Instituto Nacional de Cardiología Ignacio Chávez, Mexico City, Mexico; ^2^Coronary Care Unit, Instituto Nacional de Cardiología Ignacio Chávez, Mexico City, Mexico

**Keywords:** STEMI, vasculitis, polyarteritis nodosa, myocardial infarction, vasospasm (VS)

## Abstract

This report describes a rare case of a global myocardial infarction caused by severe vasospasm of the coronary arteries secondary to the administration of pyridostigmine in a patient with polyarteritis nodosa (PAN). Details about the clinical presentation, the typical electrocardiographic pattern of multivessel disease, the differential diagnoses suspected in the multi-imaging approach, and the treatment of cardiogenic shock are described. The definitive diagnosis of infarction and the histopathological findings compatible with polyarteritis nodosa were made by autopsy.

## Introduction

Polyarteritis nodosa (PAN) is a systemic vasculitis that typically affects medium-sized vessels (1). Coronary arteries are less frequently affected, and symptoms of myocardial ischemia and infarction are rare (2).

We present the case of a 53-year-old female patient referred to the emergency department for suspected acute coronary syndrome. While her coronary angiography was normal, she rapidly developed refractory cardiogenic shock and died. Autopsy revealed a massive global myocardial infarction and findings consistent with polyarteritis nodosa. To our knowledge, this is the first case of acute global myocardial infarction in a patient with polyarteritis nodosa. The catastrophic presentation of this case is the result of the combination of an underlying disease and precipitant factors.

### Case description

**FIGURE 1:**
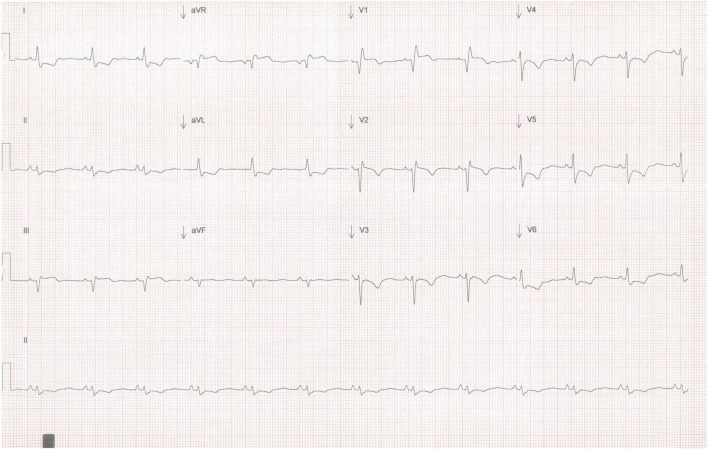
Twelve-lead EKG showing high-risk ischemic features. ST-segment elevation is observed in aVR and V1, as well as ST-segment depression in six different leads (V3, V4, V5, V6, DI, aVL).

A 53-year-old female patient with no comorbidities or previous clinical history began 6 months before her admission to the emergency department (ED) with weakness of lower extremities. She consulted a neurologist who, suspecting myasthenia gravis, prescribed pyridostigmine and prednisone, however, no improvement was observed. A few days after the initiation of pyridostigmine, the patient suffered sudden onset of chest pain and presented to the ED. Vital signs were: 110/80 mmHg, heart rate 86 beats per minute, respiratory rate 18 breaths per minute and physical examination was normal on admission. An electrocardiogram demonstrated ST-segment elevations in aVR and V1, in addition to ST-segment depressions from V3 to V6, DI and aVL (Figure 1). High-sensitivity cardiac troponin T level were 9164 ng/l (0–8 ng/l). Thus, non-ST segment elevation myocardial infarction (NSTEMI) was diagnosed.

### Diagnostic assessment

**FIGURE 2:**
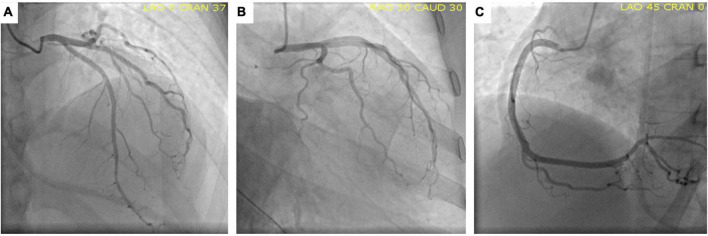
Normal coronary angiography. Anterior-posterior cranial view **(A)**, Caudal left-anterior-oblique view **(B)** and Left-anterior-oblique view **(C)** showing the circumflex artery, left anterior descending artery, and right coronary artery, respectively, without lesions.

Since a non-ST segment elevation myocardial infarction was the first suspected diagnosis, the patient was taken to the cardiac catheterization laboratory, where the coronary angiogram revealed no significant lesions (Figure 2).

According to the diagnostic approach for MINOCA, additional coronary angiography review by several experts was performed to ensure the absence of obstructive coronary artery disease, and to rule out intracoronary emboli/thrombi and spontaneous coronary artery dissection (3). Intracoronary imaging and intracoronary physiological tests were not incorporated in the approach since no obstructive coronary lesions were found in the context of acute coronary syndrome.

**FIGURE 3:**
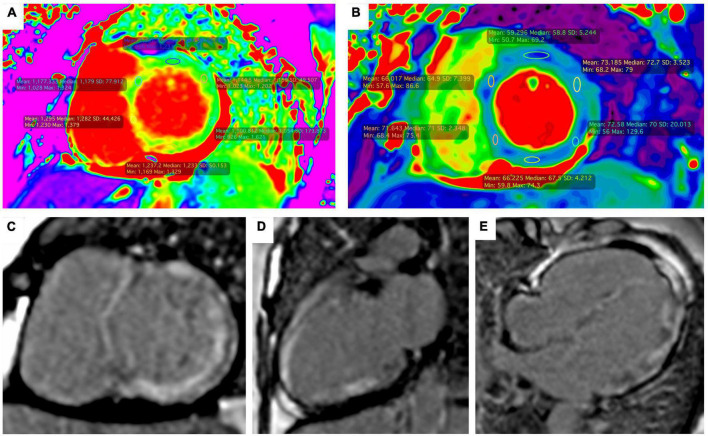
Magnetic resonance imaging. Short-axis ShMOLLI color maps showing diffuse high native values T1 **(A)** and T2 **(B)** which is compatible with myocardial edema and fibrosis. Late gadolinium enhancement with intramyocardial and transmural pattern was observed in the short axis view **(C)**, two chambers view **(D)** and four chambers view **(E)**.

Transthoracic echocardiogram demonstrated global wall-motion abnormalities; global thickening of the left ventricle and severe systolic dysfunction (LVEF 20%); findings suggestive of differential diagnoses such as Takotsubo cardiomyopathy or other cardiomyopathies were excluded. Cardiac magnetic resonance imaging (MRI) showed late enhancement with gadolinium distributed globally and heterogeneously throughout the left ventricle (Figure 3).

Intramuscular electromyography exhibited prolonged latencies, reduced amplitudes, absence of F waves and absence of H reflex were observed, findings consistent with polyradiculoneuropathy.

### Therapeutic interventions

Shortly after hospital admission, the patient showed hemodynamic deterioration. Blood pressure dropped to 70/50 mmHg and cold extremities, profuse sweating, and dyspnea were noted. Due to the fact that the hemodynamic evaluation showed a low cardiac index, high pulmonary artery wedge pressure and high peripheral vascular resistance, the diagnosis of cardiogenic shock was made. Norepinephrine and dobutamine infusions were started to maintain peripheral perfusion.

Based on the significant yet inconclusive MRI findings, endomyocardial biopsy was conducted in order to more accurately distinguish between myocarditis and myocardial infarction. Unfortunately, the biopsy was inconclusive, demonstrating non-specific inflammation. In the subsequent hours, hypotension and low cardiac index persisted. Levosimendan drip was started, elective endotracheal intubation was performed, and an intra-aortic balloon pump was placed as bridge to bridge treatment.

### Follow up and outcomes

Despite hemodynamic support, and while waiting for ECMO cannulation, the patient developed refractory cardiac arrest. After 30 min of resuscitation, the medical team together with the patient’s family decided to stop CPR due to poor prognosis.

**FIGURE 4:**
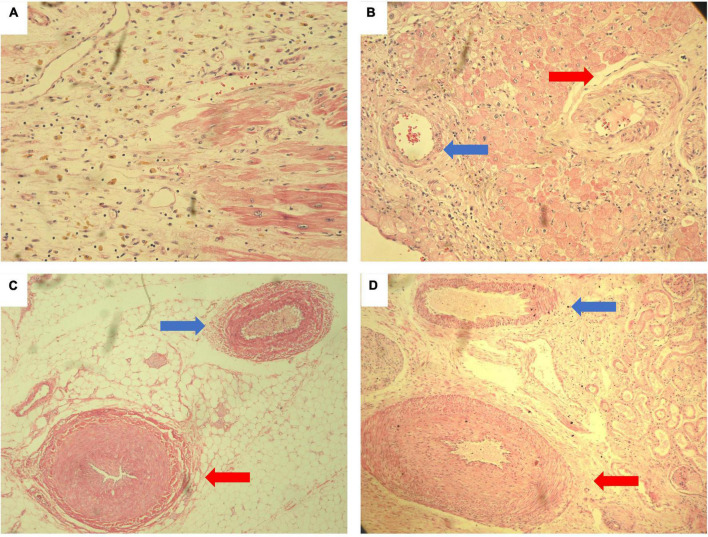
Histological autopsy samples stained with hematoxylin and eosin. Myocardial necrosis with reparative granulation tissue formed by fibroblasts with initial deposits of collagen fibers. Numerous pigmented macrophages (whose cytoplasm is reddish) and abundant new vessels. Findings compatible with acute myocardial infarction of 10 to 14 days of evolution. **(A)** Intramyocardial coronary artery with marked thickening of the wall and reduced lumen (red arrow). There are spindle cells with the appearance of reparative fibroblasts that cause a disorganized appearance for comparison. On the left (blue arrow), an artery with normal characteristics is observed. **(B)** Medium caliber mesenteric artery (red arrow) with marked thickening of the wall that reduces the lumen to a cleft. In comparison, a mesenteric artery with normal characteristics is observed (blue arrow). **(C)** Medium caliber renal artery with marked thickening of the wall due to intimal hyperplasia that significantly reduces the lumen of the vessel (red arrow). Above (blue arrow) a renal artery with normal characteristics **(D)**.

An autopsy was performed to identify the underlying cause of the cardiogenic shock and consequently the cause of death. An infarct of variable thickness (transmural and non-transmural) was found throughout the entire left ventricle, in addition to intimal thickening in the coronary arteries. These findings were also found in kidney and intestine arteries, which in conjunction with the muscle weakness secondary to polyneuritis led to the diagnosis of Polyarteritis nodosa (Figure 4).

## Discussion

In contrast to the myocardial infarction in patients with atherosclerotic disease, most of the infarction events reported in patients with PAN are related to the disease pathology itself: up to 13% of patients may have coronary lesions such as stenosis, aneurysms, spontaneous coronary dissection or ectasia (4). These characteristic coronary dilations produce a vascular bed where blood flow may be slow or turbulent which in turn may facilitate the formation of thrombi, occlusion and subsequent infarction (5).

Previously, only one case of myocardial infarction in a patient with PAN and normal coronary arteries has been previously reported; however, features of such case included a well-located myocardial territory and a mild clinical presentation (6) which differ from the present report.

In our case, the diagnosis of probable PAN was based on the Japanese diagnostic criteria, revised in 2005 (7), meeting 2 clinical criteria (myocardial infarction and polyneuritis), angiographic findings (occlusion of the bowel arteries). Although no fibrinoid necrosis was found in the biopsies, the involvement of medium and small caliber arteries was confirmed, without involvement of arterioles, venules or capillaries, which is typical in PAN (8). Historical findings suggest that the arteries were in a chronic phase of the disease where fibrinoid necrosis is replaced by marked intimal thickening with apparent obliteration of the lumen with minimal or no cellular swelling around the walls of the arteries (9).

Beyond the alterations described in the arteries, the definitive diagnosis of infarction was confirmed at autopsy. This was key for the description of the pathophysiology of cardiac involvement and in order to differentiate other entities, such as myocarditis, given the cardiac magnetic resonance findings.

Although the definitive diagnosis of myocarditis remains histopathological, it is true that cardiac magnetic resonance findings may be sufficient to establish a strong suspicion of this disease (10). However, there are multiple entities such as myocarditis, myocardial infarction, amyloidosis, focal or diffuse fibrosis whose characteristics overlap in cardiac magnetic resonance findings (11). In fact, large studies comparing the characteristics of acute myocardial infarction and myocarditis on cardiac magnetic resonance base the differences on the pattern of late enhancement (transmural, subepicardial, and subendocardial) and whether the distribution corresponds to a vascular territory (12). In our case, the affected regions of the myocardium did not correspond to a single vascular territory, so the suspicion of an infarction practically vanished at that time.

It was not until the autopsy that the diagnosis of myocarditis was ruled out. Multiple histological sections were made and none of them met the Dallas diagnosis criteria for myocarditis (13). Instead, an infiltrate characterized by neutrophils, granulation tissue with the formation of new blood vessels and collagen deposition was observed. Typical of acute myocardial infarction with an evolution of 10 to 14 days (14). Same time of evolution of the clinical picture of the patient.

Although the distribution of the infarct does not obey a specific vascular territory, non-transmural multifocal diffuse infarcts have been described as a well-established cause of MINOCA, especially under pathophysiological substrates such as diffuse vasospasm (15).

The authors hypothesized a vasospastic component for the present case. According to Lanza et al., two factors are required for this hypothesis to be true: a susceptible vessel and a triggering stimulus. Susceptibility to vasospasm is related to microstructural changes caused by chronic inflammation, fibromuscular hyperplasia and adventitial abnormalities, presents in PAN, which cause vessel hyperreactivity (16). While the triggering stimuli of vasospasm seem to be related to sympathetic and parasympathetic activity (17). In this particular case, we suspect that the stimulus that could trigger spasm in all the coronary arteries could have been the pyridostigmine that he received before the onset of his clinical presentation (18). Under normal conditions, acetylcholine causes vasodilation through the secretion of nitric oxide in the parasympathetic nerves. However, in high doses, it can induce direct stimulation of muscarinic receptors and cause vasoconstriction.

It is possible that the greatest limitation in this approach is not having any imaging test that demonstrates coronary vasospasm. Nevertheless, it is possible to postulate the association since the patient had the substrate to develop vasospasm (alterations in the coronary arteries due to vasculitis) and she received a stimulus that has already been reported as a cause of vasospasm (pyridostigmine) (18). The timing of the onset of symptoms also supports the suspicion, since it was not until the patient received pyridostigmine that she began with chest pain and later cardiogenic shock. Finally, histopathology gave the definitive diagnosis of myocardial infarction and did not show occlusions or thrombi in the coronary arteries.

This case constitutes another type of coronary artery disease manifestation described so far in patients with PAN. It strengthens the theory that the ischemic substrate can be caused by coronary vasospasm and exemplifies that in such patients, myocardial infarctions not limited to a vascular territory can also occur and cause serious hemodynamic consequences.

## Data availability statement

The original contributions presented in this study are included in the article/supplementary material, further inquiries can be directed to the corresponding author.

## Author contributions

ER-M, JL-L, FV-S, CC-G, AG-O, EA-C, and JV-E wrote the clinical case. FS-J, DG, RG-N, AA-M, JB, and DM wrote the discussion. All authors contributed to the article and approved the submitted version.
